# Native shrub densities predict burrow co-occurrence patterns in Central California Drylands

**DOI:** 10.1186/s12862-024-02259-6

**Published:** 2024-05-24

**Authors:** Ethan Owen, Christopher J. Lortie, Mario Zuliani

**Affiliations:** Department of Biological Science, York University, 4700 Keele St, Toronto, ON M3J 1P3 Canada

**Keywords:** Burrow, Shrub density, Desert, Foundational species, Resource

## Abstract

Ecological resource availability is crucial for the persistence and survival of local desert animal communities. Dryland resources such as shrubs and burrows positively benefit animal species by mitigating harsh abiotic factors and providing habitat. Understanding the role of native shrubs, many of which serve as foundation species within desert regions, as well as the function of underground burrows as resources, provides insights into habitat utilization. In this study, we seek to better understand the co-occurrence of these two resources as a first step in quantifying key patterns locally and regionally in drylands. We tested whether the presence of burrows increased with the density of foundational shrubs near the burrows at two scales—within a 5 m radius of every burrow recorded and at the site level—defined as discrete ecological areas. We performed fieldwork across 31 sites within the arid and semiarid regions of Central California. We used a combination of burrow field surveys and satellite imagery to document both vertebrate animal burrow frequencies and shrub densities. Additionally, the accuracy of the shrub data was verified through ground truthing. Both fine-scale and site-level shrub densities positively predicted the relative likelihood of burrows and the frequency of burrows, respectively. The existence of two highly utilized dryland resources and the relationship between them signal that areas abundant in both resources will likely better support resident animal species. This finding underscores the significance of incorporating both shrub density and burrow frequency in studies of habitat interconnectivity and quality. The co-occurrence patterns of these resources will support novel habitat management and conservation strategies designed around both conservation and restoration efforts.

**Supplementary Information:**

The online version contains supplementary material available at 10.1186/s12862-024-02259-6.

## Introduction

Global dryland ecosystems are fundamentally shaped by semifossorial herbivorous mammals, many of which are considered ecosystem engineers and have a variety of positive impacts on the surrounding arid region [1, 2]. These impacts that ecosystem engineers exert on their environment often have a disproportionate influence on the patterns of species diversity in their communities [3, 4]. Utilizing burrows is a common behavioral adaptation for animal species that occur in dryland regions across the globe, as it can provide animals with several benefits, including a sheltered microclimate, protection from predators, and access to food [5–8]. In dryland ecosystems, these burrows provide many local species with a more favorable microclimate by buffering temperature extremes and increasing relative humidity, thus creating a more suitable microclimate [9–11]. Often found in large communal colonies, burrowing mammals can transform dryland regions through the creation of complex underground burrows and feeding activity patterns [12–15]. These burrows range from microsite-level to extensive regional-level systems, resulting in major impacts on soil dynamics, vegetation patterns and animal community diversity, ultimately increasing heterogeneity across a region [1, 9, 10, 16, 17]. Within southern California, the giant kangaroo rat (*Dipodomys ingens)* is a state and federally endangered species of burrowing rodent [18]. The extensive network of burrows that giant kangaroo rats construct provides habitat for the federally endangered blunt-nosed leopard lizard (*Gambelia sila)* and the state-threatened San Joaquin antelope squirrel (*Ammospermophilus nelsoni)* [19–21]. Giant kangaroo rats are also the primary prey source for the endangered San Joaquin kit fox (*Vulpes macroitis mutica)* and thus play an integral role in the structure and function of the ecosystem [18].

Rodent burrows serve as pivotal hubs for their activities within the region. Essential for the survival of small vertebrate species that occur in harsh arid environments, these burrows serve as a crucial resource, providing refuge from extreme temperatures and predators. As focal points for rodent activity, burrows influence the spatial distribution of vegetation, particularly in their immediate vicinity [3]. The relative frequency of rodent activity decreases with increasing distance from these burrows, leading to compositional heterogeneity in vegetation, which tends to increase closer to burrows [17]. This heightened diversity and variability in plant species abundance and distribution are attributed to localized effects such as seed dispersal, soil disturbance, and nutrient cycling facilitated by rodent activity [15, 18]. Understanding these spatial patterns of burrowing rodent activity and their interplay with other local landscape resources is crucial for elucidating the intricate dynamics within dryland regions.

Vegetation is an important part of any region because it can not only influence local vertebrate and invertebrate communities but also shape the structure of an ecosystem [22, 23]. Positive interactions associated with local vegetation are incredibly important in resource-limited environments, such as those found in dryland regions [24]. Dominant shrub species in arid environments are often beneficial to a wide range of animal and plant species, resulting in frequent positive interactions [24, 25]. In dryland regions, facilitation often occurs when certain shrubs create microclimates that provide shade, trap moisture, and offer protection, thus promoting the survival and growth of neighboring plant and animal species [22, 26]. This mutualistic interaction is beneficial for the environment because it enhances biodiversity, stabilizes ecosystems, and contributes to the overall resilience of desert regions by fostering conditions conducive to plant and animal life despite the challenging arid environment [16, 22, 27, 28]. In dryland regions, it has been shown that heterogeneous shrub density can benefit local vertebrate and invertebrate species, thus increasing habitat heterogeneity across arid regions [20, 24, 29]. The increase in shrub availability has the potential to provide more possible interactions for local species, thus increasing the frequency of facilitative interactions [8, 30]. A better understanding of how both burrows created by local vertebrates and naturally occurring foundational shrub species are spatially related will provide meaningful insight into how local animal communities utilize regional resources to persist in harsh arid environments.

The benefits associated with foundational shrub species within dryland ecosystems can promote the association of burrowing vertebrate species [24, 31]. These association can drive an increase in the abundance of burrows around shrub individuals [32]. In addition, these burrowing vertebrate species can disperse seeds throughout the landscape, promoting shrub growth at burrow areas [33]. This suggests that there should be an association between shrub densities and burrow abundance within dryland ecosystems. As these dryland ecosystems experience drier and higher temperatures associated with increasing aridity, the dependency on these foundational shrub species can potentially increase [30]. The benefits through shading and the microclimate produced from these shrubs can ameliorate these increasingly arid conditions [30, 31].

While previous studies have examined the independent utilization of burrows and foundation shrubs by local desert animal communities, there remains a gap in understanding the spatial association between these resources at both fine and site-level scales [24, 34, 35]. Here, we tested the hypothesis that there is a relationship between the fine-scale and site-level density estimates of the foundational shrub species *E. californica* and *L. tridentata* and the presence and relative frequencies of burrows formed by vertebrate animal species.

We tested the following predictions:

- The relative frequency of burrows will increase total shrub density at each site.
- Increasing relative aridity will increase the effects of shrub density on the total burrows per site.
- The likelihood of burrow presence increases with fine-scale shrub density (i.e., within a 5 m radius, as tested here).

## Methods

### Species and Study sites

**Fig. 1:**
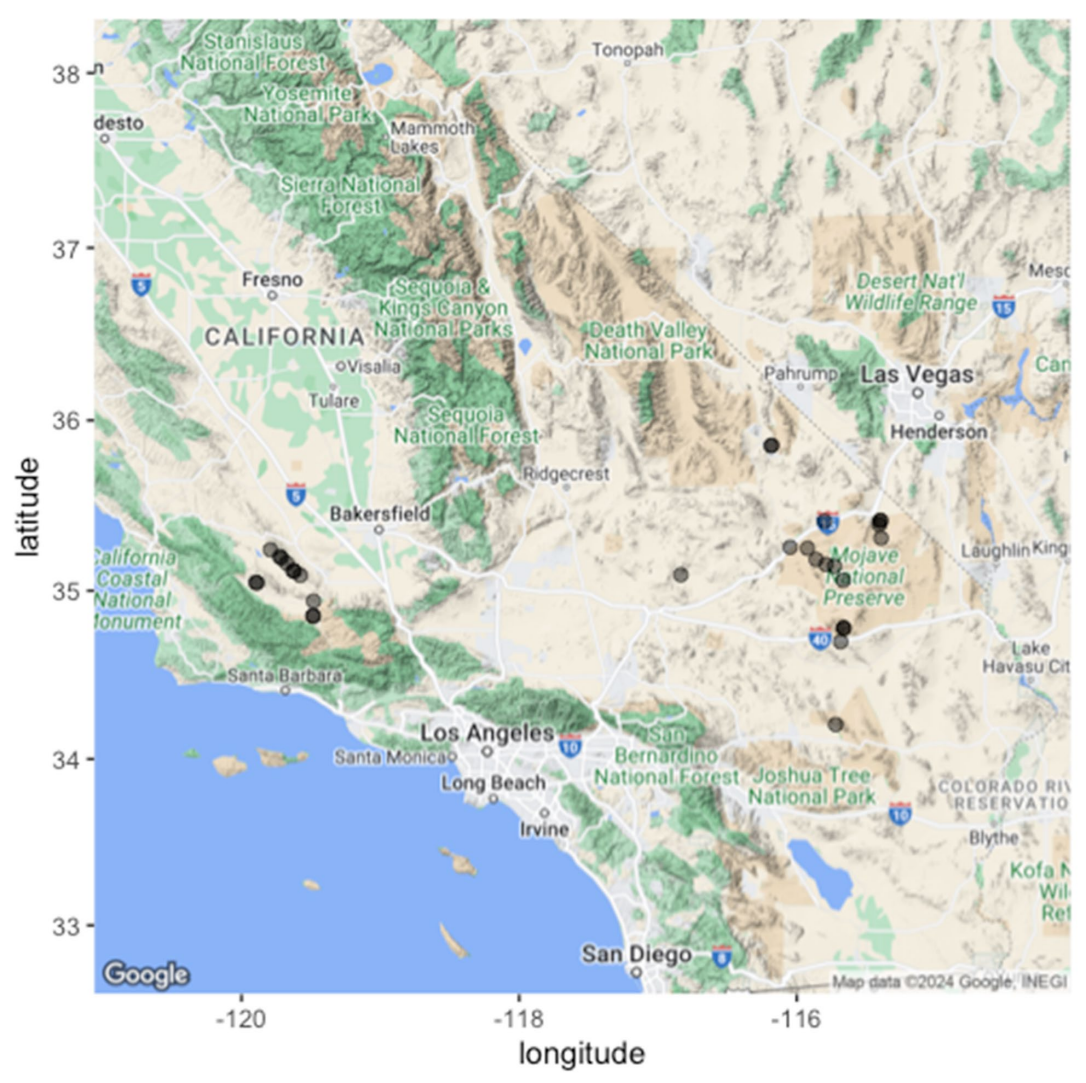
Geographical representation of all study sites within southern California, U.S.A. A total of 30 unique sites were utilized for burrow and shrub data collection through the 2023 field season. All sampled sites are indicated with black circles. Random samples of burrows were taken at all 30 sites where the presence and density of shrubs were recorded via satellite imagery. The map was created through R version 4.3.1

A total of 30 sites were established across southern California on an east-to-west gradient (Fig. 1; Supplementary Table S1) [36]. Key variables describing sites included foundational shrub species, geographic location, mean annual temperature (MAT), mean annual precipitation (MAP), and estimated shrub density (Supplementary Table S1). These variables were used to select and differentiate sites used within this study. A total of 30 sites were used to encompass a gradient of climate and shrub densities ranging from 0 to 166 shrubs per site (Supplementary Table S1). All sites were dominated by either the woody shrub species *Ephedra californica* or *Larrea tridentata* (Supplementary Figure S1 & S2) [8, 37, 38]. Both shrub species function as ecological foundational species for both plant [26, 38, 39] and animal communities by increasing species abundance and richness of many taxa [20, 24, 40]. These species include small to medium-sized vertebrates, including antelope squirrels (*Ammospermophilus* sp.) kangaroo rats (*Dipodomys* sp.), blunt-nosed leopard lizards (*Gambelia sila)*, and black-tailed jack rabbits (*Lepus californicus*) [24, 41–51]. Gradients of these foundational shrubs across California influence the associations of local animal community distributions and composition [24, 30, 46].

### Site-level shrub density effects on total burrows

The annual aridity of each site was determined using the De Martonne aridity index equation AI = P/(MAT + 10) where P represents the annual precipitation in mm within 20 years and MAT represents the mean annual temperature (°C) within 20 years [52]. These climate data were retrieved from the WorldClim version 2.1 database at a 1 km spatial resolution [52, 53]. Shrub density estimates for each of the 30 sites were derived from remotely sensed imagery. Specifically, *Ephedra californica* and *Larrea tridentata* density were estimated using Google Earth composite satellite images [8]. The base layer of these maps retrieved by google were sourced by the Airbus Earth Observation Satellite Imagery Services with a spatial resolution of 30 cm [54]. All individual shrubs at each site were geolocated and given a unique identifier. Once all *E. californica* and *L. tridentata* individuals were identified, a series of keyhole markup language files (KMLs) were extracted. The latitude and longitude coordinates for each shrub were reported [55, 56]. A total of 200 random points were ground-truthed at one of the thirty sites to confirm the accuracy of the satellite-based shrub density measures within a 5-meter plot in field [57]. Total burrows per site were then calculated for site level density effects using the detailed field survey protocol described below.

### Fine-scale analysis of shrub density effects on burrows

Burrow surveys were done at the same 30 sites from May to June of 2023. A single 25 m x 25 m quadrat was randomly established at each site. The quadrat was used to visually count burrows created by vertebrates. Coordinates (decimal degrees) were collected at each independent burrow opening using a handheld GPS (Garmin-GPSMAP65). Shrub density within a 5 m radius was estimated for each burrow within the 25 m x 25 m quadrate by combining in field burrow observations with the satellite shrub data. Although the resolution of burrow points and shrubs can vary between 3 m and 5 m, potentially leading to false positive associations [8], our analysis explored associations within various radii including 5 m, 10 m, and 20 m. We tested theses associations at 5 m, 10 m, and 20 m radii for all 30 sites. The observed patterns remain robust across these different spatial scales. Pseudoabsence points were spatially generated using a random sampling approach using the R package *sf* [58]. Field observations were then used to ensure burrow openings were correctly attributed to vertebrates (Supplementary Figure S4 & S5). Vertebrate burrows typically display visible tracks or nearby feces, that the current pattern use from invertebrate burrows (Supplementary Figures S3, S4, & S5). To further eliminate the possible misclassification during burrow surveys, instances where burrows exhibited clear indications of frequent or recent rodent activity were recorded along with detailed characteristics [59]. These data are published alongside all other attributes recorded [59]. Burrow diameters were measured and ranged from 4.4 cm to 11.2 cm. Vertebrate animal responsible for burrow formations were not identified.

### Statistical analysis

#### Site-level shrub density effects on total burrows

A second-order polynomial regression model was used to test the effect of site-level shrub density on the total number of burrows recorded per site [60]. Total number of burrows per site was the response variable, and total shrub density per site was the main factor. Aridity was also examined as a factor. All assumptions of regression were tested for the site-level analysis including multicollinearity using the R package ‘*performance’* [61].

### Fine-scale analysis of shrub density effects on burrow

The relative importance of shrub density on predicted presence of burrows at fine-scales, i.e. within a 5 m radius of each burrow, was tested with a general linear mixed model with binary data - otherwise known as a logistic regression [62]. Since the response variable within the models was represented by binary data, the presence or absence of a shrub, a logistic regression best estimates the probability of the observations belonging to one of the two categories [62]. The presence-absence of burrows was the response variable while shrub density within 5 m of each burrow was the factor, nested within sites. This presence-absence data were modelled as binomial [63]. The package ‘*aod*’ was used [64] to calculate the predicted probabilities of burrows at the range of shrub densities calculated. The null likelihood and full model with shrub density effects at probability of burrows were contrasted using the ‘*performance*’ R package function *test_performance* [65, 66]. The probability of burrow presences and absence range based on certainty and do not necessarily assume up to 1 or 100% [67]. All statistical analyses and models were done in R version 4.3.1 [68].

## Results

### Site-level shrub density effects on total burrows

**Fig. 2:**
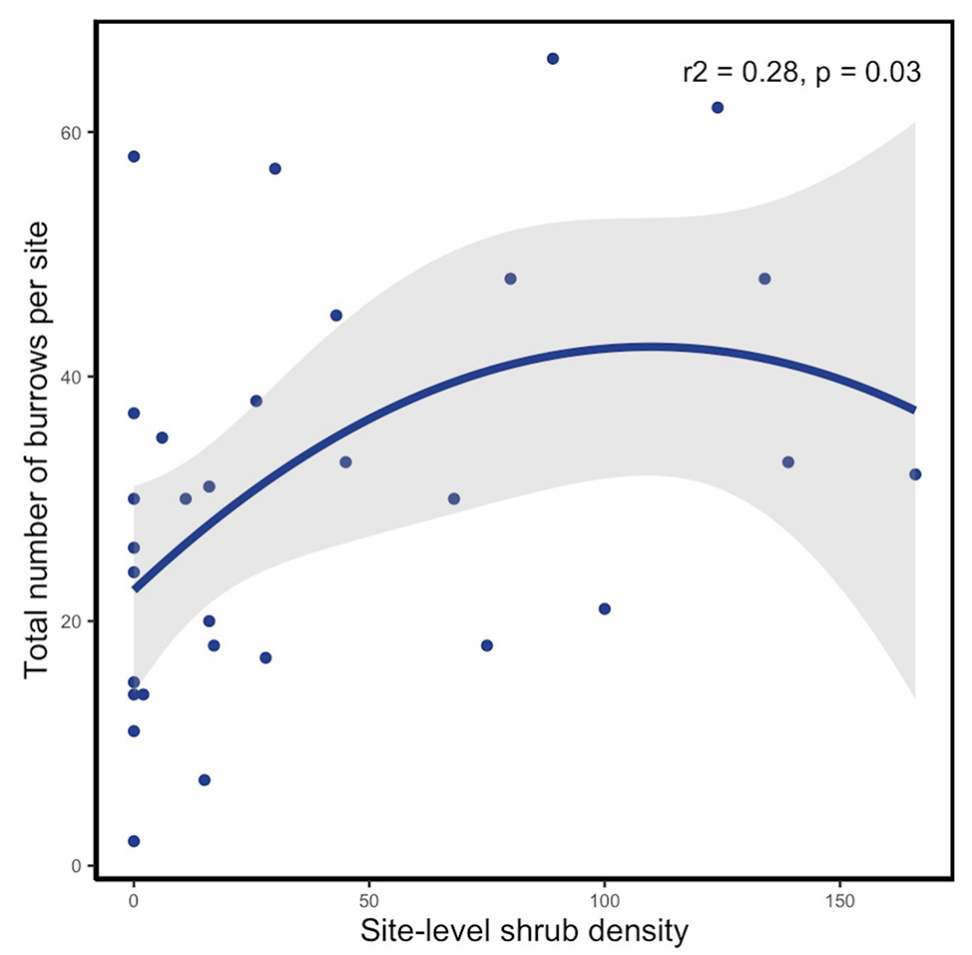
The effect of shrub density on the total number of burrows across Central California desert sites. The total count of burrows observed in the field surveys was regressed against the total shrub density per site from satellite imagery data (r2 = 0.28, p value = 0.03)

There were no statistically significant differences between the geolocated shrub density estimates and the ground-truthed, field shrub density counts (Supplement Table S2; paired t-test, t = -0.05, df = 389.41, *p* = 0.96). Total shrub density per site significantly predicted the total number of burrows (Fig. 2; regression, R^2^ = 0.28, df = 26, p-value = 0.03). Increasing aridity decreased the total number of burrows per site (estimate 21.1, df = 27, p-value = 0.001), but aridity across sites did not significantly influence the positive effects of shrub density on burrows (estimate = 8.09, *df* = 26, p-value = 0.15). Regressions to explore shrub densities within 10 m and 20 m of burrows per site expressed similar trends (Supplement Table S3).

### Fine-scale analysis of shrub density effects on burrows

**Fig. 3:**
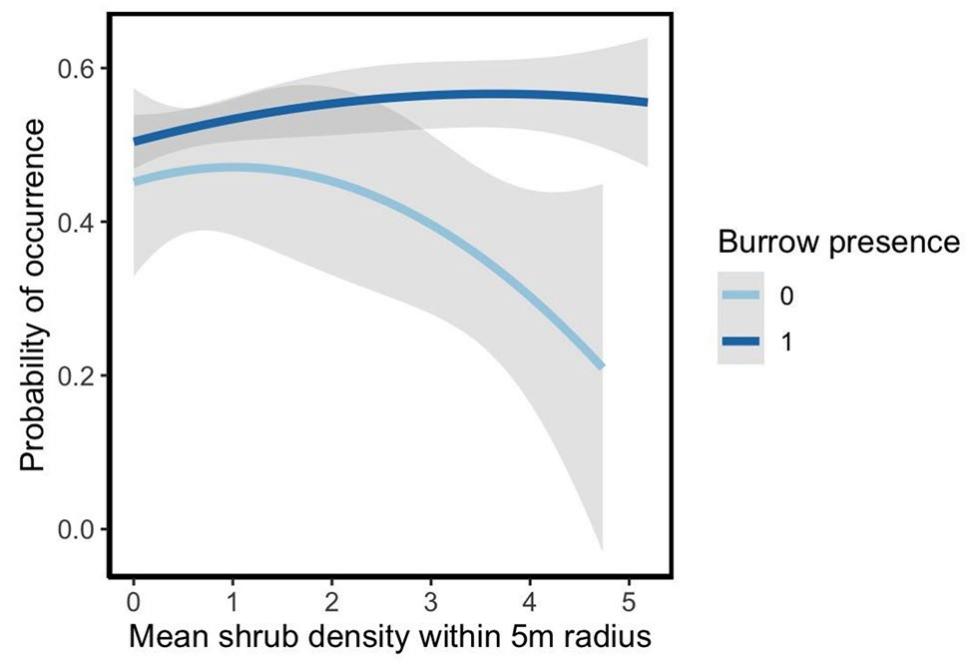
The predicted probabilities of burrows with increasing shrub density within a 5 m radius. The presence of a burrow is shown in dark blue (1), and the absence of a burrow is shown in light blue (0). Shaded areas show the 95% confidence intervals for each line of best fit

The probability of burrow presence significantly increased with increasing shrub density within a 5 m radius of each observed instance (Fig. 3; GLMM, estimate = 161.46, *df* = 26, p value = 0.001). The predictive model nested by site performed significantly better than the non-nested model (GLM; ω^2^ = 0.04, LR = 161.46, p-value < 0.001).

## Discussion

Exploring the co-occurrence patterns between burrow abundance and shrub density can reveal some of the key forms of heterogeneity in resources within dryland regions. Here, we tested whether there is a relationship between the density of foundational shrub species and the presence of burrows across various regions within Central California. We found evidence supporting the hypothesis that sites with foundational shrubs exhibit a significantly high number of burrows. Shrub density was greater around burrows than in areas where burrows were absent, and increasing shrub density significantly predicted a greater probability of burrow presence.

Physical structure can influence ecological processes in a variety of complex ways, influencing how local species communities form, function, and persist [69, 70]. Burrow systems are more than just ‘holes in the ground’; they are a critical resource for many species [18, 21, 71]. Burrowing mammals generally have a positive impact on species richness at the regional level by increasing habitat diversity [10, 71]. The potential consequences of losing burrowing mammals in desert regions extend beyond a mere disruption of biodiversity; they may have a disproportional impact on the distribution of other organisms and the overall functioning of desert ecosystems. In particular, the absence of burrow systems could exacerbate temperature extremes and sun exposure in arid environments, creating unfavorable conditions for various species [1, 19, 30]. Moreover, the combined threats of wildfires, desertification, a decline in shrub health, and the negative stigma of shrub encroachment pose additional risks to the vertebrate populations inhabiting these regions [72–74]. These challenges will lead to disruptions in behavioral patterns, migration routes, and reproductive success, ultimately posing a threat to the ecological balance of these ecosystems [2, 10]. Understanding the multifaceted role of burrow systems is therefore essential for comprehending their broader implications for regional dynamics and biodiversity conservation.

Increasing annual temperatures during summer are a major issue for vertebrates in desert regions [75]. When faced with continuous solar radiation and high temperatures, some species of mammals visit cooler microclimates to reduce their overall exposure [74.75]. The dehydration effect of dry desert air on life forms is a second significant barrier to survival in desert and arid regions [6]. The microclimate of subterranean rodent burrows has been shown to be much more favorable than that on the surface because the relative humidity inside burrows is near saturation [6, 12, 71]. Ecosystem engineers that construct these site-level burrows can directly influence plant communities through a variety of mechanisms and thus play integral roles in shaping the surrounding vegetation and ecosystem dynamics [1, 13, 75]. *Dipodomys* sp. in California maintain circles of bare ground around their burrows, creating open spaces lacking annual plants [18, 71, 76]. They have also been known to inadvertently transport seeds within their fur or bury them in caches near their burrows, facilitating the dispersal and germination of plant species across the region [75, 77, 78]. The intricate design and network of these burrows exert substantial influence on the surrounding environment, shaping both direct and indirect impacts on local animal and plant communities [75, 77]. On a regional scale, the presence of burrowing animals and burrows can promote biodiversity by providing habitat and resources for a wide range of plant and animal species. Additionally, the ecosystem engineering effects of burrowing animals contribute to nutrient cycling, soil aeration, and water infiltration, thereby influencing the resilience and functioning of dryland regions [71, 79]. Foundational shrubs typically benefit animal communities in dryland regions through a variety of mechanisms, including providing a refuge from harsh temperatures, serving as a food source for some small animals, and preventing predation [8, 22, 80]. Zuliani et al. [24] reported greater numbers of burrowing mammals, such as *Dipodomys* sp. and *Ammospermophilus* sp., in areas with greater shrub densities, supporting the idea that smaller vertebrate species are reliant on the positive effects of foundational shrubs.

Although not explicitly examined in this research, one of the primary functions of burrows and shrubs is to provide shelter from intense temperatures for various animal species [20, 30]. Several studies have reported greater associations of animals near shrubs during peak times of the day, suggesting that thermal amelioration is a direct benefit to these species [22, 30, 81]. This positive association of animal species with shrubs can help us explain the greater probability of burrows being observed, as several burrowing species are more frequently observed in these areas. This further supports our findings that increasing shrub density can predict the probability of burrows being present. Having a burrow within proximity to a shrub allows for many benefits, such as acting as a refuge from predation [30, 48]. In the event of a predation attempt, the presence of both a burrow and shrub can allow for either resource to be utilized as a means of escape [21, 25, 40]. It also allows for closer proximity to a food source and the ability to spend time above or below ground while still being proximal to one’s home [71]. The intricate relationships between foundational shrubs, burrowing species, and their surrounding environment highlight the significance of structural landscape features in shaping species associations and interactions in arid regions [82]. Understanding the multifaceted benefits, ranging from thermal amelioration to refuge from predation, underscores the pivotal role that increasing shrub density plays in predicting the presence of burrows.

The presence of two key desert habitat resources, shrubs and burrows, shows that both can potentially benefit local animal species [83–85] provided that we deepen our understanding and empirical knowledge of co-occurrence patterns. These data will inform habitat conservation and restoration efforts. With desertification affecting at-risk and endangered species, these findings could help guide conservation efforts by emphasizing the importance of animal burrows and the facilitative interactions between foundational shrubs and target animal species [57, 86]. As these arid regions continue to deteriorate overall ecosystem health, it is critical to investigate and include other regional resources that influence the associations between plant and animal communities. These discoveries may offer valuable perspectives on safeguarding critical habitats in ecosystems that are essential for endangered species. This insight can facilitate targeted species rehabilitation with minimal human intervention, as land managers and restoration biologists can leverage these findings to enhance the habitat association of the targeted species.

### Electronic supplementary material

Below is the link to the electronic supplementary material.

Supplementary Material 1

## Data Availability

All Burrow data is available at https://doi.org/10.6084/m9.figshare.22272622.v5.
