# Supplementary material for: Native shrub densities predict burrow co-occurrence patterns in Central California Drylands
Source: BMC Ecol Evol. 2024 May 24;24:68. doi: 10.1186/s12862-024-02259-6 (PMC11118996; doi:10.1186/s12862-024-02259-6)

**Supplements**

**Table S1:** Site-level data corresponding to each site where burrow data were collected. The elevation is given in meters, the mean annual temperature (MAT) is given in °C, the mean annual precipitation (MAP) is given in mm, and the aridity was calculated using the De Martonne aridity index and the mean values for 20 years of long-term data from WorldClim. Shrub density is the total number of shrubs in a 0.25 km2 area derived from satellite imagery, and total burrow counts are the counts of burrows in plots from the field surveys done in 2023.

| site | foundation shrub species | burrowing vertebrate species | long | lat | elevation | MAT | MAP | aridity | Shrub density | Total burrows |
| --- | --- | --- | --- | --- | --- | --- | --- | --- | --- | --- |
| Carrizo_1 | Ephedra californica | San Joaquin Antelope Squirrel, California ground squirrel, Burrowing Owl, Blunt-Nosed Leopard Lizard, Kangaroo rat, San Joaquin Kit Fox, America Badger | -119.72495 | 35.20177 | 726 | 14.3 | 150 | 6.2 | 17 | 18 |
| Carrizo_2 | Ephedra californica |  | -119.71253 | 35.19266 | 756 | 14.3 | 150 | 6.2 | 6 | 35 |
| Carrizo_3 | Ephedra californica |  | -119.67445 | 35.16245 | 749 | 14.5 | 149 | 6.0 | 2 | 14 |
| Carrizo_4 | Ephedra californica |  | -119.62069 | 35.11566 | 716 | 14.7 | 147 | 5.9 | 30 | 57 |
| Carrizo_6 | Ephedra californica |  | -119.79147 | 35.24177 | 670 | 14.6 | 155 | 6.3 | 28 | 24 |
| Carrizo_7 | Ephedra californica |  | -119.80846 | 35.2473536 | 650 | 14.8 | 157 | 6.3 | 20 | 17 |
| Carrizo_soda_open | open |  | -119.57727 | 35.0906062 | 740 | 14.6 | 146 | 5.9 | 0 | 58 |
| Carrizo_soda_shrub | Ephedra californica |  | -119.62974 | 35.1192136 | 701 | 14.7 | 148 | 5.9 | 0 | 37 |
| Cuyama_1 | Ephedra californica |  | -119.48312 | 34.848726 | 848 | 14.0 | 136 | 5.7 | 11 | 30 |
| Cuyama_2 | Ephedra californica |  | -119.48596 | 34.853622 | 837 | 14.1 | 137 | 5.7 | 16 | 20 |
| Cuyama_3 | Ephedra californica |  | -119.48084 | 34.938244 | 827 | 14.2 | 139 | 5.7 | 124 | 62 |
| Cuyama_4 | Ephedra californica |  | -119.48049 | 34.947224 | 838 | 14.2 | 139 | 5.7 | 22 | 14 |
| Cuyama_5 | Ephedra californica |  | -119.8891 | 35.045952 | 557 | 14.8 | 151 | 6.1 | 80 | 48 |
| Cuyama_6 | Ephedra californica |  | -119.89618 | 35.049012 | 504 | 15.1 | 152 | 6.1 | 26 | 38 |
| Barstow_1 | Ephedra californica | Mojave Ground Squirrel, Desert Kangaroo Rat, White-tailed Antelope Squirrel, Round-tailed Ground Squirrel, Burrowing Owl | -116.8349 | 35.094051 | 590 | 19.3 | 160 | 5.5 | 33 | 33 |
| Barstow_2 | Ephedra californica |  | -117.98 | 35.02 | 800 | 16.6 | 160 | 6.0 | 30 | 26 |
| Heartofmojave | Ephedra californica |  | -115.68417 | 34.698199 | 784 | 19.7 | 142 | 4.8 | 89 | 66 |
| Kelso_1 | Larrea tridentata |  | -115.40502 | 35.3889 | 917 | 18.2 | 136 | 4.8 | 0 | 15 |
| Kelso_2 | Larrea tridentata |  | -115.39279 | 35.31008 | 1048 | 17.5 | 132 | 4.8 | 134 | 48 |
| Kelso_3 | Larrea tridentata |  | -115.40158 | 35.41113 | 919 | 18.0 | 137 | 4.8 | 139 | 33 |
| Kelso_4 | Larrea tridentata |  | -115.39379 | 35.41392 | 877 | 18.3 | 137 | 4.8 | 100 | 21 |
| Kelso_5 | Larrea tridentata |  | -115.66436 | 35.061279 | 781 | 19.4 | 140 | 4.7 | 0 | 30 |
| Mojave_1 | Larrea tridentata |  | -116.04723 | 35.2560833 | 313 | 21.6 | 158 | 5.0 | 68 | 30 |
| Mojave_2 | Larrea tridentata |  | -115.92243 | 35.2502 | 515 | 20.5 | 153 | 5.0 | 75 | 18 |
| Mojave_3 | Larrea tridentata |  | -115.86002 | 35.1842833 | 737 | 19.4 | 148 | 5.0 | 15 | 7 |
| Mojave_4 | Larrea tridentata |  | -115.79033 | 35.1551667 | 951 | 18.0 | 140 | 4.9 | 166 | 32 |
| Mojave_5 | Larrea tridentata |  | -115.72862 | 35.1445333 | 1128 | 17.1 | 135 | 4.9 | 16 | 31 |
| Sheephole | Ephedra californica |  | -115.71968 | 34.205676 | 545.92 | 31.0 | 152 | 4.8 | 43 | 45 |
| Tecopa | Ephedra californica | Mojave Ground Squirrel, Desert Kangaroo Rat, White-tailed Antelope Squirrel, Round-tailed Ground Squirrel, Burrowing Owl, Amargosa Vole | -116.18671 | 35.851515 | 453 | 20.1 | 165 | 5.5 | 0 | 26 |
| Tecopa_open | open |  | -116.17866 | 35.85474 | 465 | 20.1 | 165 | 5.5 | 0 | 2 |

**Table S2:** The comparison of satellite measured shrub density and corresponding ground-truthed data, collected at 1 of the sampled sites. 200 random locations were selected in one field site shrub density was ground-truthed.

| rep | latitude | longitude | shrub_density | ground_truth |
| --- | --- | --- | --- | --- |
| 1 | 35.110358 | -119.629134 | 3 | 4 |
| 2 | 35.111187 | -119.623025 | 3 | 3 |
| 3 | 35.111191 | -119.622806 | 2 | 4 |
| 4 | 35.110518 | -119.628927 | 3 | 3 |
| 5 | 35.111196 | -119.622586 | 3 | 3 |
| 6 | 35.110527 | -119.628489 | 6 | 6 |
| 7 | 35.111367 | -119.623031 | 1 | 2 |
| 8 | 35.111372 | -119.622811 | 20 | 16 |
| 9 | 35.110674 | -119.630029 | 11 | 12 |
| 10 | 35.110712 | -119.628275 | 3 | 4 |
| 11 | 35.110727 | -119.627617 | 3 | 3 |
| 12 | 35.110731 | -119.627398 | 2 | 2 |
| 13 | 35.111376 | -119.622592 | 12 | 12 |
| 14 | 35.110765 | -119.625863 | 6 | 5 |
| 15 | 35.110769 | -119.625644 | 0 | 0 |
| 16 | 35.111381 | -119.622373 | 4 | 4 |
| 17 | 35.111552 | -119.622817 | 5 | 5 |
| 18 | 35.110845 | -119.630473 | 13 | 13 |
| 19 | 35.11085 | -119.630254 | 12 | 12 |
| 20 | 35.111556 | -119.622598 | 12 | 13 |
| 21 | 35.111561 | -119.622379 | 1 | 3 |
| 22 | 35.111523 | -119.63247 | 20 | 19 |
| 23 | 35.111737 | -119.622604 | 2 | 2 |
| 24 | 35.111741 | -119.622384 | 1 | 1 |
| 25 | 35.111812 | -119.619096 | 13 | 15 |
| 26 | 35.111873 | -119.63292 | 16 | 17 |
| 27 | 35.110949 | -119.62565 | 3 | 4 |
| 28 | 35.110968 | -119.624773 | 4 | 4 |
| 29 | 35.110973 | -119.624554 | 3 | 3 |
| 30 | 35.112044 | -119.633364 | 0 | 0 |
| 31 | 35.112215 | -119.633808 | 0 | 0 |
| 32 | 35.111025 | -119.630479 | 9 | 10 |
| 33 | 35.11103 | -119.63026 | 8 | 7 |
| 34 | 35.111049 | -119.629383 | 10 | 10 |
| 35 | 35.111058 | -119.628944 | 7 | 7 |
| 36 | 35.111082 | -119.627848 | 8 | 8 |
| 37 | 35.112224 | -119.63337 | 0 | 0 |
| 38 | 35.112229 | -119.63315 | 0 | 0 |
| 39 | 35.111106 | -119.626752 | 0 | 0 |
| 40 | 35.111111 | -119.626533 | 1 | 1 |
| 41 | 35.112238 | -119.632712 | 0 | 0 |
| 42 | 35.112414 | -119.632937 | 0 | 0 |
| 43 | 35.111153 | -119.62456 | 5 | 5 |
| 44 | 35.112633 | -119.622852 | 0 | 0 |
| 45 | 35.112704 | -119.619563 | 5 | 6 |
| 46 | 35.112822 | -119.614082 | 42 | 32 |
| 47 | 35.112818 | -119.622638 | 4 | 4 |
| 48 | 35.112788 | -119.632291 | 0 | 0 |
| 49 | 35.112988 | -119.623083 | 21 | 24 |
| 50 | 35.113002 | -119.622425 | 6 | 5 |
| 51 | 35.113059 | -119.619794 | 2 | 3 |
| 52 | 35.113173 | -119.622869 | 12 | 13 |
| 53 | 35.113239 | -119.6198 | 4 | 4 |
| 54 | 35.111243 | -119.628731 | 6 | 8 |
| 55 | 35.113244 | -119.61958 | 0 | 0 |
| 56 | 35.113367 | -119.61388 | 9 | 11 |
| 57 | 35.113329 | -119.623971 | 3 | 3 |
| 58 | 35.113348 | -119.623094 | 2 | 2 |
| 59 | 35.111305 | -119.625881 | 2 | 2 |
| 60 | 35.111315 | -119.625442 | 0 | 0 |
| 61 | 35.111324 | -119.625004 | 3 | 3 |
| 62 | 35.111334 | -119.624565 | 4 | 4 |
| 63 | 35.111357 | -119.623469 | 1 | 1 |
| 64 | 35.111395 | -119.621715 | 0 | 0 |
| 65 | 35.111405 | -119.621277 | 1 | 1 |
| 66 | 35.113367 | -119.622217 | 11 | 14 |
| 67 | 35.113382 | -119.621559 | 57 | 53 |
| 68 | 35.111361 | -119.631587 | 15 | 13 |
| 69 | 35.113386 | -119.62134 | 56 | 32 |
| 70 | 35.11341 | -119.620244 | 0 | 0 |
| 71 | 35.111395 | -119.630052 | 13 | 13 |
| 72 | 35.11342 | -119.619805 | 14 | 12 |
| 73 | 35.111419 | -119.628956 | 12 | 15 |
| 74 | 35.111423 | -119.628737 | 17 |  |
| 75 | 35.111452 | -119.627421 | 4 | 4 |
| 76 | 35.111457 | -119.627202 | 4 | 4 |
| 77 | 35.113557 | -119.613447 | 0 | 0 |
| 78 | 35.113562 | -119.613228 | 0 | 0 |
| 79 | 35.113543 | -119.622442 | 10 | 10 |
| 80 | 35.11149 | -119.625667 | 1 | 1 |
| 81 | 35.111504 | -119.62501 | 2 | 2 |
| 82 | 35.111518 | -119.624352 | 6 | 9 |
| 83 | 35.111528 | -119.623913 | 3 | 4 |
| 84 | 35.111537 | -119.623475 | 3 | 3 |
| 85 | 35.113552 | -119.622004 | 33 | 32 |
| 86 | 35.113562 | -119.621565 | 14 | 15 |
| 87 | 35.113567 | -119.621346 | 16 | 17 |
| 88 | 35.111518 | -119.632689 | 17 | 18 |
| 89 | 35.111546 | -119.631373 | 15 | 15 |
| 90 | 35.113571 | -119.621127 | 17 | 18 |
| 91 | 35.113595 | -119.620031 | 4 | 6 |
| 92 | 35.113709 | -119.614769 | 4 | 6 |
| 93 | 35.111594 | -119.629181 | 13 | 13 |
| 94 | 35.113732 | -119.613672 | 0 | 0 |
| 95 | 35.111618 | -119.628085 | 8 | 8 |
| 96 | 35.113666 | -119.625079 | 13 | 12 |
| 97 | 35.111684 | -119.625015 | 2 | 2 |
| 98 | 35.111699 | -119.624358 | 1 | 1 |
| 99 | 35.111703 | -119.624138 | 3 | 3 |
| 100 | 35.113723 | -119.622448 | 5 | 5 |
| 101 | 35.111775 | -119.62085 | 1 | 2 |
| 102 | 35.111784 | -119.620411 | 0 | 0 |
| 103 | 35.113728 | -119.622229 | 6 | 6 |
| 104 | 35.111808 | -119.619315 | 16 | 17 |
| 105 | 35.111688 | -119.633133 | 6 | 6 |
| 106 | 35.111698 | -119.632695 | 14 | 15 |
| 107 | 35.111703 | -119.632475 | 20 | 21 |
| 108 | 35.111731 | -119.63116 | 6 | 6 |
| 109 | 35.113733 | -119.62201 | 1 | 1 |
| 110 | 35.113742 | -119.621571 | 1 | 1 |
| 111 | 35.113747 | -119.621352 | 16 | 19 |
| 112 | 35.111784 | -119.628748 | 14 | 14 |
| 113 | 35.111798 | -119.628091 | 9 | 9 |
| 114 | 35.113756 | -119.620913 | 44 | 40 |
| 115 | 35.111812 | -119.627433 | 8 | 8 |
| 116 | 35.111822 | -119.626994 | 2 | 3 |
| 117 | 35.11377 | -119.620256 | 8 | 8 |
| 118 | 35.113775 | -119.620036 | 6 | 7 |
| 119 | 35.113898 | -119.614336 | 4 | 3 |
| 120 | 35.111841 | -119.626117 | 0 | 0 |
| 121 | 35.113903 | -119.614117 | 8 | 8 |
| 122 | 35.11185 | -119.625679 | 3 | 3 |
| 123 | 35.113908 | -119.613897 | 2 | 2 |
| 124 | 35.113917 | -119.613459 | 0 | 0 |
| 125 | 35.113879 | -119.62355 | 8 | 10 |
| 126 | 35.113889 | -119.623112 | 16 | 17 |
| 127 | 35.113894 | -119.622892 | 48 | 44 |
| 128 | 35.113898 | -119.622673 | 10 | 10 |
| 129 | 35.111917 | -119.622609 | 1 | 1 |
| 130 | 35.111926 | -119.622171 | 1 | 1 |
| 131 | 35.111936 | -119.621733 | 0 | 0 |
| 132 | 35.113903 | -119.622454 | 0 | 0 |
| 133 | 35.111959 | -119.620636 | 0 | 0 |
| 134 | 35.113932 | -119.621138 | 0 | 0 |
| 135 | 35.113936 | -119.620919 | 2 | 2 |
| 136 | 35.113941 | -119.6207 | 18 | 15 |
| 137 | 35.112002 | -119.618663 | 1 | 2 |
| 138 | 35.113946 | -119.620481 | 9 | 11 |
| 139 | 35.111864 | -119.633358 | 0 | 0 |
| 140 | 35.111869 | -119.633139 | 2 | 2 |
| 141 | 35.111883 | -119.632481 | 13 | 16 |
| 142 | 35.111911 | -119.631166 | 7 | 7 |
| 143 | 35.111926 | -119.630508 | 4 | 5 |
| 144 | 35.111931 | -119.630289 | 5 | 6 |
| 145 | 35.113955 | -119.620042 | 40 | 34 |
| 146 | 35.111964 | -119.628754 | 10 | 10 |
| 147 | 35.114064 | -119.614999 | 0 | 0 |
| 148 | 35.114088 | -119.613903 | 0 | 0 |
| 149 | 35.112002 | -119.627 | 0 | 0 |
| 150 | 35.113865 | -119.632545 | 0 | 0 |
| 151 | 35.114003 | -119.626187 | 7 | 7 |
| 152 | 35.114012 | -119.625748 | 5 | 5 |
| 153 | 35.114017 | -119.625529 | 6 | 6 |
| 154 | 35.114036 | -119.624652 | 12 | 13 |
| 155 | 35.11406 | -119.623556 | 30 | 33 |
| 156 | 35.114069 | -119.623117 | 12 | 13 |
| 157 | 35.114074 | -119.622898 | 7 | 8 |
| 158 | 35.114079 | -119.622679 | 0 | 0 |
| 159 | 35.114098 | -119.621802 | 0 | 0 |
| 160 | 35.114102 | -119.621583 | 0 | 0 |
| 161 | 35.114136 | -119.620048 | 0 | 0 |
| 162 | 35.114145 | -119.619609 | 6 | 6 |
| 163 | 35.114154 | -119.619171 | 12 | 15 |
| 164 | 35.114254 | -119.614567 | 0 | 0 |
| 165 | 35.114207 | -119.625096 | 5 | 7 |
| 166 | 35.114245 | -119.623342 | 10 | 10 |
| 167 | 35.114249 | -119.623123 | 2 | 2 |
| 168 | 35.11433 | -119.619396 | 8 | 10 |
| 169 | 35.114335 | -119.619177 | 18 | 20 |
| 170 | 35.114349 | -119.618519 | 8 | 9 |
| 171 | 35.114387 | -119.616765 | 1 | 1 |
| 172 | 35.114415 | -119.615449 | 1 | 2 |
| 173 | 35.11442 | -119.61523 | 2 | 3 |
| 174 | 35.114377 | -119.625541 | 3 | 3 |
| 175 | 35.114396 | -119.624664 | 7 | 8 |
| 176 | 35.114415 | -119.623787 | 24 | 23 |
| 177 | 35.110523 | -119.628708 | 4 | 4 |
| 178 | 35.111191 | -119.631143 | 19 | 21 |
| 179 | 35.111224 | -119.629608 | 16 | 15 |
| 180 | 35.111262 | -119.627854 | 12 | 12 |
| 181 | 35.111352 | -119.632025 | 18 | 20 |
| 182 | 35.111476 | -119.626325 | 1 | 1 |
| 183 | 35.111604 | -119.620406 | 1 | 1 |
| 184 | 35.111551 | -119.631154 | 9 | 9 |
| 185 | 35.111565 | -119.630496 | 13 | 15 |
| 186 | 35.11158 | -119.629839 | 14 | 12 |
| 187 | 35.111584 | -119.629619 | 11 | 11 |
| 188 | 35.111642 | -119.626989 | 4 | 5 |
| 189 | 35.111746 | -119.630502 | 11 | 11 |
| 190 | 35.111826 | -119.626775 | 4 | 5 |
| 191 | 35.111879 | -119.624363 | 2 | 2 |
| 192 | 35.111964 | -119.620417 | 0 | 0 |
| 193 | 35.111997 | -119.618882 | 3 | 3 |
| 194 | 35.110835 | -119.630912 | 11 | 12 |
| 195 | 35.11084 | -119.630692 | 12 | 11 |
| 196 | 35.110916 | -119.627185 | 7 | 8 |
| 197 | 35.110983 | -119.624115 | 1 | 1 |
| 198 | 35.11102 | -119.630698 | 14 | 16 |
| 199 | 35.111565 | -119.630496 | 13 | 15 |
| 200 | 35.111741 | -119.622384 | 1 | 1 |

**Table S3:** Regression analysis data comparing burrow presence with increasing shrub density at 5m, 10m, and 20m radius. Model comparison suggests that all radii have similar trends.

| **Scale** | **Response** | **R^2^** | **df** | **Slope** | **p-value** | **Regression AICc** |
| --- | --- | --- | --- | --- | --- | --- |
| 5m | Number of burrows | 0.28 | 26 | 44.2 | 0.001 | 255.3 |
| 10m | Number of burrows | 0.25 | 26 | 39.15 | 0.023 | 256.5 |
| 20m | Number of burrows | 0.25 | 26 | 40.02 | 0.022 | 256.4 |

**Figure S1:** Photograph of the foundational shrub *Larrea tridentata*, commonly known as creosote bush, showcasing its characteristic resinous leaves and intricate branching pattern. Photograph taken on 15/05/2023 at the Tecopa_shrub site located in the Mojave National Preserve.


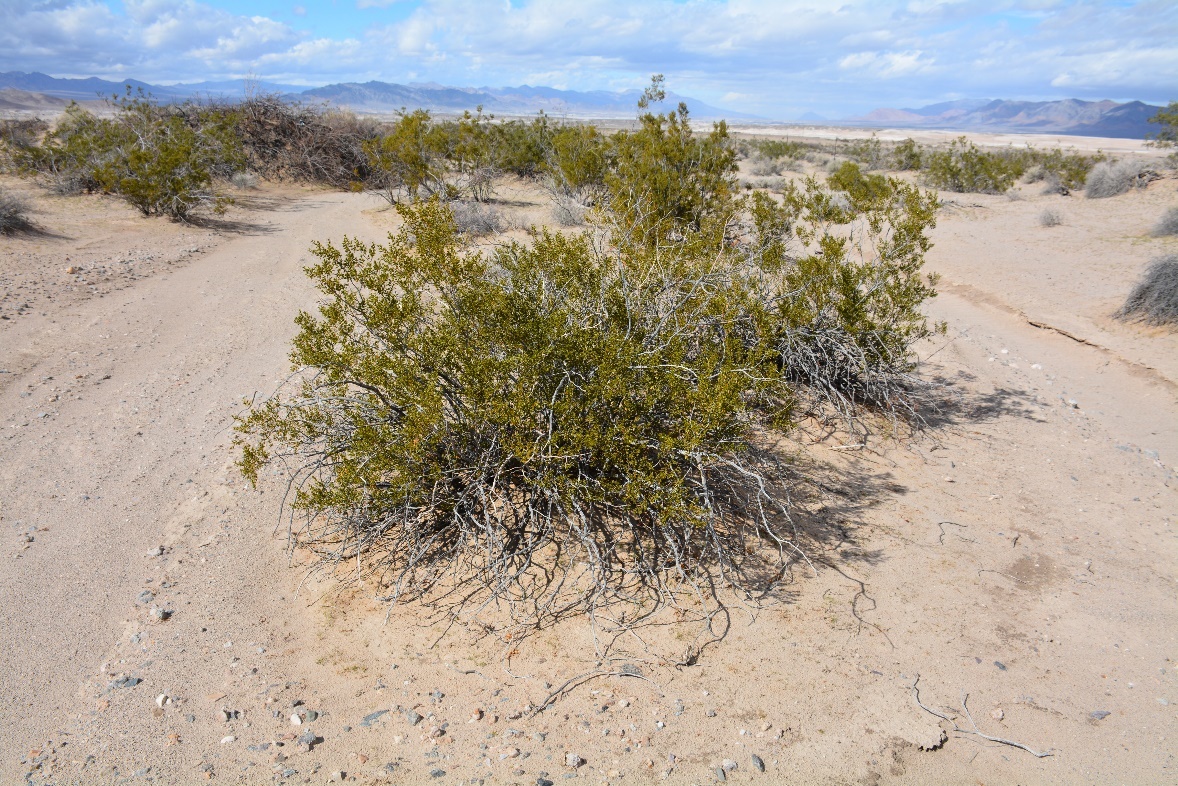


**Figure S2:** Photograph of rodent burrow entrance located near foundational shrub *Ephedra californica*, also known as California jointfir. Photograph taken on 10/06/2023 at the Carrizo_Soda_Shrub site located in the Carrizo Plain National Monument.


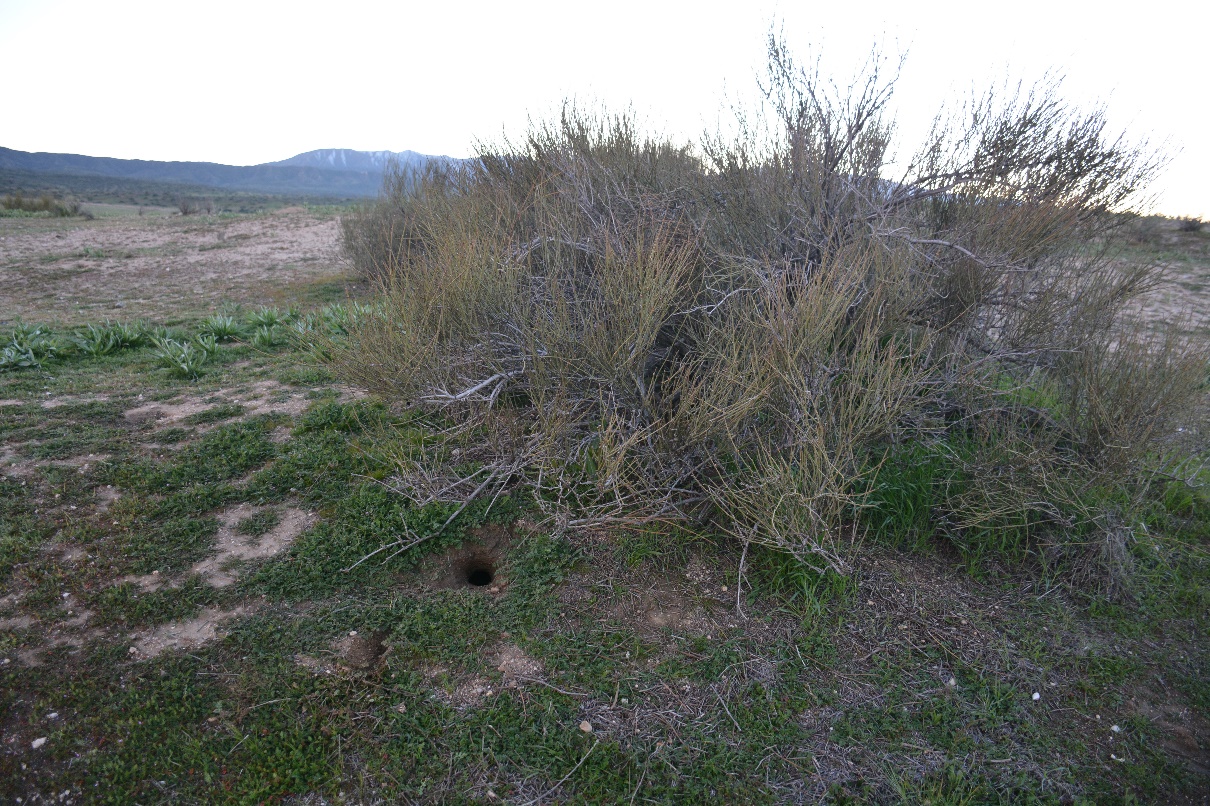


**Figure S3**: Photograph of a relatively small animal burrow entrance (5.3 cm). This burrow shows signs of recent excavation, fresh tracks and worn paths leading to and from the burrow opening. Photograph taken on 10/07/2023 at the Carrizo_3 site located in the Carrizo Plain National Monument.
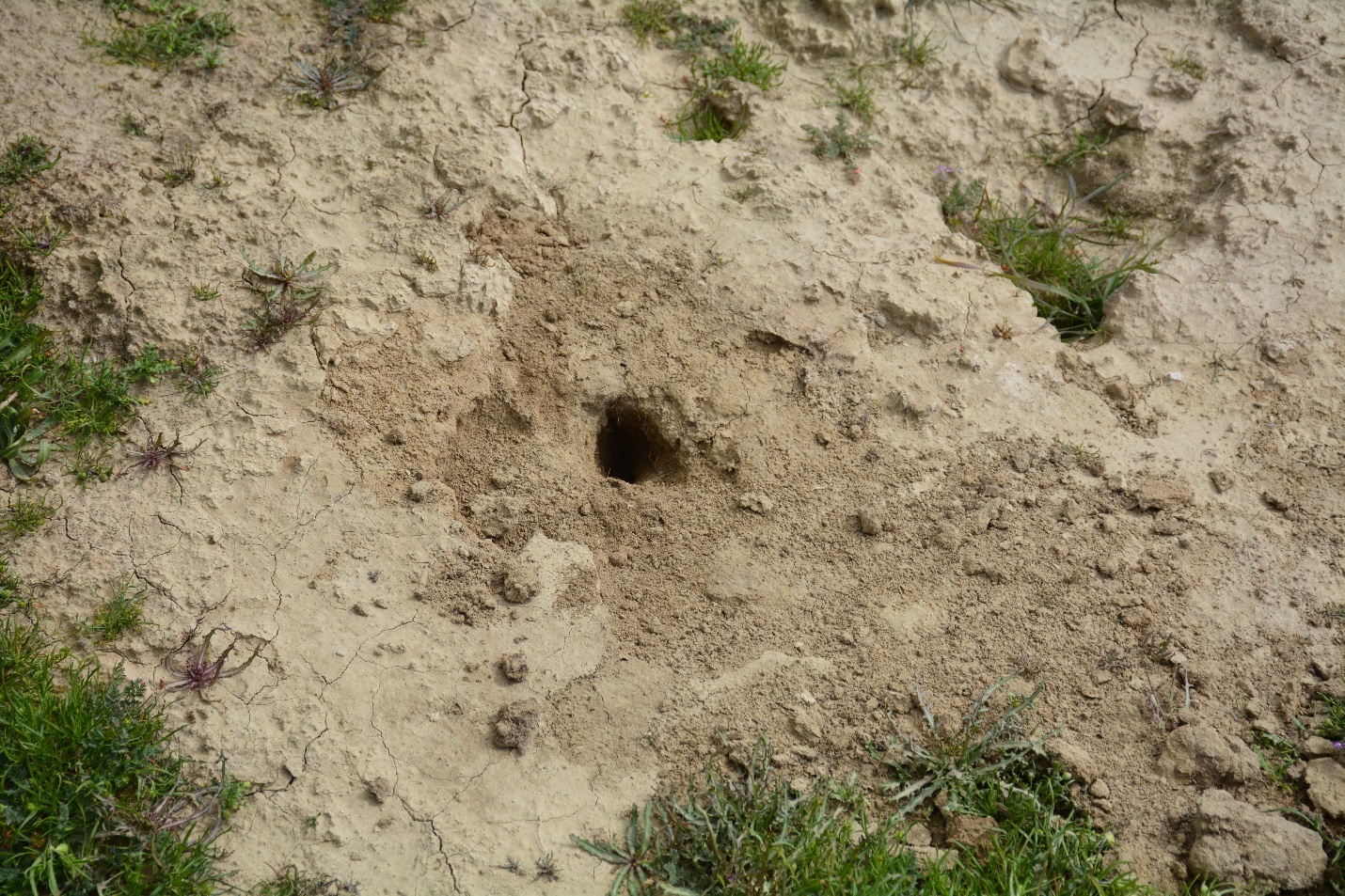


**Figure S4:** Photograph of animal burrow entrance (11.5 cm). This burrow shows strong signs of recent use including excavation, scat and worn paths leading to and from the burrow opening. Photograph taken on 10/08/2023 at the Carrizo_4 site located in the Carrizo Plain National Monument.


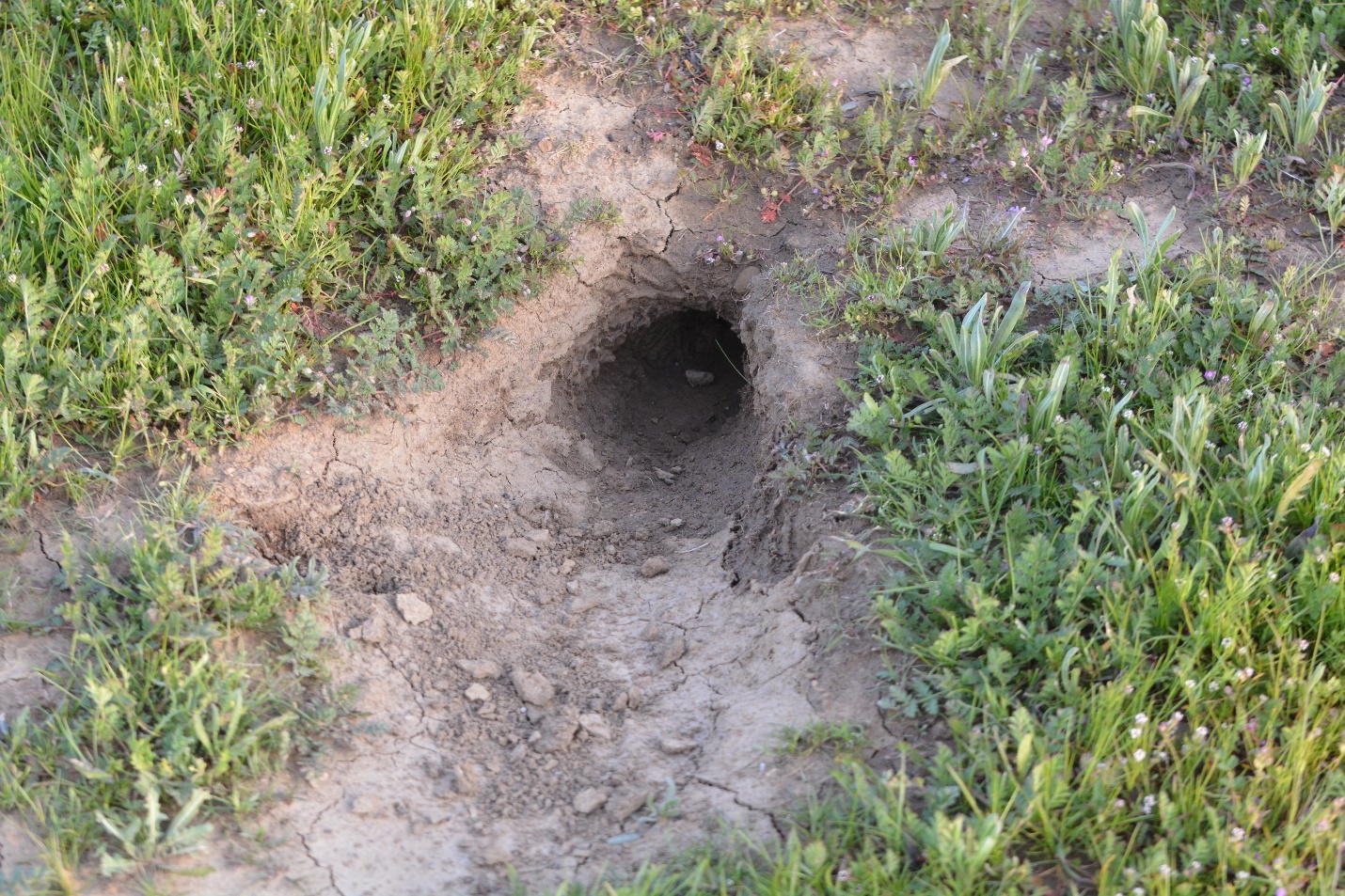

Supplement: Supplementary file 1 — Supplementary Material 1 [file 12862_2024_2259_MOESM1_ESM.docx]
